# Urolithin A Attenuates Hyperuricemic Nephropathy in Fructose-Fed Mice by Impairing STING-NLRP3 Axis-Mediated Inflammatory Response *via* Restoration of Parkin-Dependent Mitophagy

**DOI:** 10.3389/fphar.2022.907209

**Published:** 2022-06-15

**Authors:** Cong Zhang, Yingying Song, Liang Chen, Peng Chen, Ming Yuan, Yan Meng, Qi Wang, Guohua Zheng, Zhenpeng Qiu

**Affiliations:** ^1^ College of Pharmacy, Hubei University of Chinese Medicine, Wuhan, China; ^2^ Department of Pharmacy, Renmin Hospital of Wuhan University, Wuhan, China; ^3^ Key Laboratory of Chinese Medicine Resource and Compound Prescription, Ministry of Education, Hubei University of Chinese Medicine, Wuhan, China; ^4^ Hubei Key Laboratory of Resources and Chemistry of Chinese Medicine, Hubei University of Chinese Medicine, Wuhan, China

**Keywords:** urolithin A, hyperuricemic nephropathy, mitophagy, STING, NLRP3

## Abstract

Urolithin A (UroA) is one of the primary intestinal metabolites of ellagitannins, showing translational potential as a nutritional intervention in humans. Mounting evidence suggests that fructose consumption contributes to the progression of chronic kidney disease (CKD) that manifests in hyperuricemic nephropathy, renal inflammation, and tubulointerstitial injury. Here, we investigated the efficacy of UroA in alleviating fructose-induced hyperuricemic nephropathy in mice. Uric acid-exposed human kidney-2 (HK-2) cells were utilized for *in vitro* mechanism validation. Histopathological staining, immunoblotting, and transmission electron microscope were performed for the mechanistic investigations. Our results revealed that UroA ameliorated fructose-induced hyperuricemic nephropathy in mice. The histopathologic assessment showed that UroA attenuated tubular hypertrophy and dilation, glomerular basement membrane thickening, and collagen deposition in the kidney of fructose-fed mice. Mechanistically, UroA treatment impaired STING-NLRP3 activation, resulting in reduced production of proinflammatory cytokines IL-1β, IL-6, and TNF-α. Notably, UroA exhibited a scavenging effect against reactive oxygen species (ROS) and restored fructose-impaired PINK1/Parkin-mediated mitophagy in nephropathic mice. Furthermore, the inhibitory effect of UroA in STING-NLRP3 activation was impaired after Parkin gene silencing in HK-2 cells. Together, this study suggests that UroA alleviates fructose-induced hyperuricemic nephropathy by promoting Parkin-dependent mitophagy, thereby suppressing STING-NLRP3 axis-mediated inflammatory response. Thus, dietary supplementation with UroA or ellagitannins-rich foods may serve as a promising intervention to prevent CKD progression.

## Introduction

Chronic kidney disease (CKD) is a global public health issue defined as persistent alterations in kidney structure and function with adverse outcomes of end-stage renal disease (ESRD) and cardiovascular disease (CVD) (Jha et al., 2013). An epidemiology analysis indicates that the prevalence of CKD is estimated to be 700 million worldwide, with an increasing annual incidence and mortality (Collaboration, 2020). Intervention from the earlier stage of CKD appears imperative to slow its progression due to limited therapies and poor prognosis, indicating an urgent need to develop practical approaches for preventing CKD progression.

Hyperuricemic nephropathy is a common subtype of CKD and a clinical complication of hyperuricemia characterized by imbalanced purine metabolism and elevated serum uric acid levels (Li et al., 2020). The dietary fructose intake has been steadily increased over the past years in the form of sucrose or high-fructose corn syrup added to numerous manufactured foods like sugar-sweetened beverages, which is recognized as an independent risk factor contributing to the growing prevalence of metabolic diseases, especially in hyperuricemic nephropathy (Bomback et al., 2010; Johnson et al., 2010). Unlike glucose, excessive fructose consumption is dedicated to elevated serum uric acid concentration by stimulating purine metabolism, leading to the deposition of uric acid and crystals in the kidney to subsequently increase the risk of hyperuricemic nephropathy and even renal failure (Gersch et al., 2007; Srivastava et al., 2018). Mitophagy is a specific mitochondrial quality control mechanism that pertains to maintaining cellular homeostasis by selectively eliminating damaged mitochondria. Renal injury and metabolic stress in CKD are closely linked to mitochondrial dysfunction (Tang et al., 2021). During canonical PINK1/Parkin-mediated mitophagy, PINK1 is rapidly accumulated on the outer mitochondrial membrane (OMM) and recruits Parkin from the cytoplasm onto damaged mitochondria for initiating the autophagic degradation of these dysfunctional mitochondria (Matsuda et al., 2010). It has been documented that mitochondrial damage-induced mitochondrial DNA (mtDNA) leakage onto cytosol can bind with cyclic-GMP-AMP synthase (cGAS) to catalyze the synthesis of cyclic GMP-AMP (cGAMP) and subsequently promote the activation of the stimulator of interferon gene (STING), a crucial factor contributing to renal inflammation, injury, and fibrosis (Chung et al., 2019). As an essential molecule for cellular innate immune and inflammatory response, STING stimulates the activation of NOD-like receptor protein 3 (NLRP3) and promotes the release of mature inflammatory cytokines (Li et al., 2019). Further, STING exhibits proinflammatory effects in kidney diseases and is considered a promising interventional target of CKD (Gaidt et al., 2017; Maekawa et al., 2019). Thus, inhibiting STING-NLRP3 axis-mediated inflammation by promoting mitophagy may be a perspective strategy to delay CKD progression.

Ellagitannins are a family of polyphenols present in fruits and nuts exhibiting promising efficacy against metabolic disorders (Kiss and Piwowarski, 2018). As the major intestinal metabolite of ellagitannins, urolithin A (UroA) is quickly gathering attention due to similar or higher intrinsic biological effects to those of the parent compounds, such as anti-inflammatory activities and pro-autophagic effects (D'Amico et al., 2021; Hasheminezhad et al., 2022). In particular, UroA is acknowledged as a mitophagy inducer for health protection (Ryu et al., 2016; Andreux et al., 2019). It is unknown whether UroA-mediated mitophagy contributes to its efficacy in hyperuricemia-related nephropathy. In this study, the therapeutic effect of UroA was investigated in a fructose-induced hyperuricemic nephropathy mouse model. Our results demonstrate that UroA ameliorates hyperuricemic nephropathy by impairing STING-NLRP3 axis-mediated inflammatory response *via* Parkin-dependent mitophagy.

## Reagents and Methods

### Reagents

Urolithin A (UroA, purity ≥97%) was bought from Sigma-Aldrich. Fructose (purity ≥99%) was purchased from Aladdin biological technology Co., Ltd (Shanghai, China). Fetal bovine serum (FBS) and Dulbecco’s modified Eagle’s medium (DMEM) were bought from Gibco (Grand Island, NY, USA). ELISA Kits of IL-1β, IL-6, and TNF-α were purchased from Ruixin Biotechnology Co., Ltd (Quanzhou, China). All other reagents were of analytical grade.

### Mouse Monitoring

Female C57BL/6 mice (weight 18–20 g) were purchased from the Center of Experimental Animal of Hubei Province (Wuhan, Hubei, China), randomly ranged into four groups (*n* = 8). The wild-type (WT) group had free access to plain water; fructose-fed groups were given 30% fructose water freely for 8 weeks (Yang et al., 2015). Meanwhile, the administration groups were intragastrically administered with a gavage of UroA (50 or 100 mg/kg) once daily (Chen et al., 2020). All mice were euthanized at the end of animal experiments to collect kidney tissues and serum for further investigations. All involved animals were managed humanely according to protocols approved by the Institutional Animal Care and Use Committee of Hubei University of Chinese Medicine (HUCMS202106003).

### Cell Culture

Human proximal tubule cell lines HK-2 were purchased from the Shanghai Institute of Biochemistry and Cell Biology (China), maintained in DMEM, along with 10% FBS and 1% penicillin-streptomycin at 37°C and 5% CO_2_. Uric acid was used to induce a hyperuricemic nephropathy cell model followed by treatment with different concentrations of UroA (0, 10, 20, 40 μM) and harvesting for further assessment after 24 h. Parkin-siRNA (GenePharma, Suzhou, China) and transfection reagent were incubated in DMEM for 20 min at room temperature to perform transfection in HK-2 cells. The Parkin-siRNA sequence (5′ to 3’) was as follows: sense: GGA​UCA​GCA​GAG​CAU​UGU​UTT; antisense: AAC​AAU​GCU​CUG​CUG​AUC​CTT. All *in vitro* experiments were repeated at least three times.

### Measurement of the Biochemical Index and Inflammatory Factor

Urine was collected before the mice were sacrificed to determine urine protein. The blood sample was centrifuged at 3,000 rpm/min to obtain serum. Serum creatinine (Cr) and blood urea nitrogen (BUN) detections were performed to evaluate kidney function following protocols. For reactive oxygen species (ROS) detection, HK-2 cells and kidney tissues were stained with a 2′, 7′-Dichlorodihydrofluorescein diacetate (DCFH-DA) fluorescence probe using a commercial kit (Nanjing Jiancheng Bioengineering Institute, Nanjing, China). The oxidative indices were measured in kidney tissues to assess renal oxidant injury, including glutathione peroxidase (GSH-Px), total superoxide dismutase (T-SOD), and malondialdehyde (MDA). ELISA Kits of IL-6, IL-1β, and TNF-α were utilized to quantity inflammatory cytokine concentration in both serum and kidney tissues according to the manufacturer’s instructions.

### Hematoxylin-Eosin (H&E), Masson’s Trichrome Staining, and Periodic Acid-Schiff Staining

Kidney tissues were collected and fixed in 10% paraformaldehyde for more than 24 h, embedded in paraffin, and cut into 5 μm sections. For kidney histopathological examination, sections were dewaxed with xylene, rehydrated with ethanol at decreasing concentrations, and then stained with H&E, Masson, and PAS dyes, respectively. These sections were then observed and pictured under a microscope (Olympus IX 73 DP80, Tokyo, Japan).

### Real-Time Quantitative Polymerase Chain Reaction

Total RNA was extracted using a TRIzol™ Reagent from kidney tissue homogenate and then converted into complementary DNA (cDNA) using a ReverTra Ace real-time PCR-RT kit. The RT-qPCR assay was subsequently performed using FastStart universal SYBR Green Master Mix (Roche, Basel, Switzerland) on an RT-qPCR thermal cycler (ABI StepOne, NY, USA) as previously described (Liu et al., 2021). The RT-qPCR amplification program was as follows (95°C × 5 min, 40 cycles of 95°C × 15 s and 60°C × 30 s) in a 10 µl reaction mixture. GAPDH was taken as an internal control for quantitative analysis. The identified primers are listed in Supplementary Table S1.

### Mitochondrial DNA Copy Number Assay

Total DNA was extracted from HK-2 cells using a mammalian genomic DNA extraction kit (Beyotime Biotechnology). NADH dehydrogenase subunit 1 (ND1) gene encoded by mtDNA and GAPDH encoded by nuclear DNA (nDNA) were determined by RT-qPCR analysis as described previously (Du et al., 2020). The mtDNA/nDNA ratio was used to assess the relative mtDNA copy number. The primers are listed in Supplementary Table S1.

### Western Blotting and Immunofluorescence

Western blot was performed to determine protein expression of kidney tissues and HK-2 cells. Briefly, cytosolic proteins were extracted by M-PER™ Mammalian Protein extraction Reagent (Thermo Scientific, USA) from kidney tissues or HK-2 cells, and the protein concentrations were then determined using a BCA kit. The extracted proteins were separated using SDS–polyacrylamide gel electrophoresis (SDS-PAGE) and then transferred onto PVDF membranes at semi-dry conditions. After blocking skim milk, the blots were incubated with primary antibodies (Supplementary Table S2) overnight and subsequently bound with corresponding second antibodies for 1 h. The immunoblotting of target proteins was visualized with ECL reagents and captured with a chemiluminescence imager (G: BOX Chemi XRQ; Syngene, Cambridge, UK). For immunofluorescent analysis, kidney paraffin sections were heated at 60°C for 45 min, then dewaxed and rehydrated using a series of washing solutions to perform antigen retrieval in boiling sodium citrate buffer. Cell climbing slides were fixed with 4% paraformaldehyde for 20 min and permeabilized with 0.3% Triton x-100/PBS for 15 min at room temperature. Prepared kidney tissue sections and fixed cell climbing slices were incubated with primary antibodies overnight at 4°C. After that, slides were washed three times with PBS, incubated with DAPI and FITC-conjugated second antibodies for 1.5 h at 37°C, and ultimately observed under a fluorescent microscope (Olympus IX 73 DP80, Tokyo, Japan).

### Transmission Electron Microscope Analysis

Kidney tissues were wholly immersed in an electron microscope fixative solution. Subsequently, the skinny slices were collected, fixated, dehydrated, infiltrated, embedded, stained, and observed by TEM (JEM1400, JEOL Ltd., Tokyo, Japan).

### Statistical Analysis

Statistical analysis was realized by Prism 7.0 (GraphPad Software Inc., San Diego, CA, USA). All data were expressed as Mean ± S.D. values. Two-tailed unpaired *t*-test and ANOVA were implemented between two groups and among three or more groups, respectively. *p* < 0.05 means statistical significance.

## Results

### Urolithin A (UroA) Ameliorates Fructose-Induced Hyperuricemic Nephropathy in Mice

**FIGURE 1:**
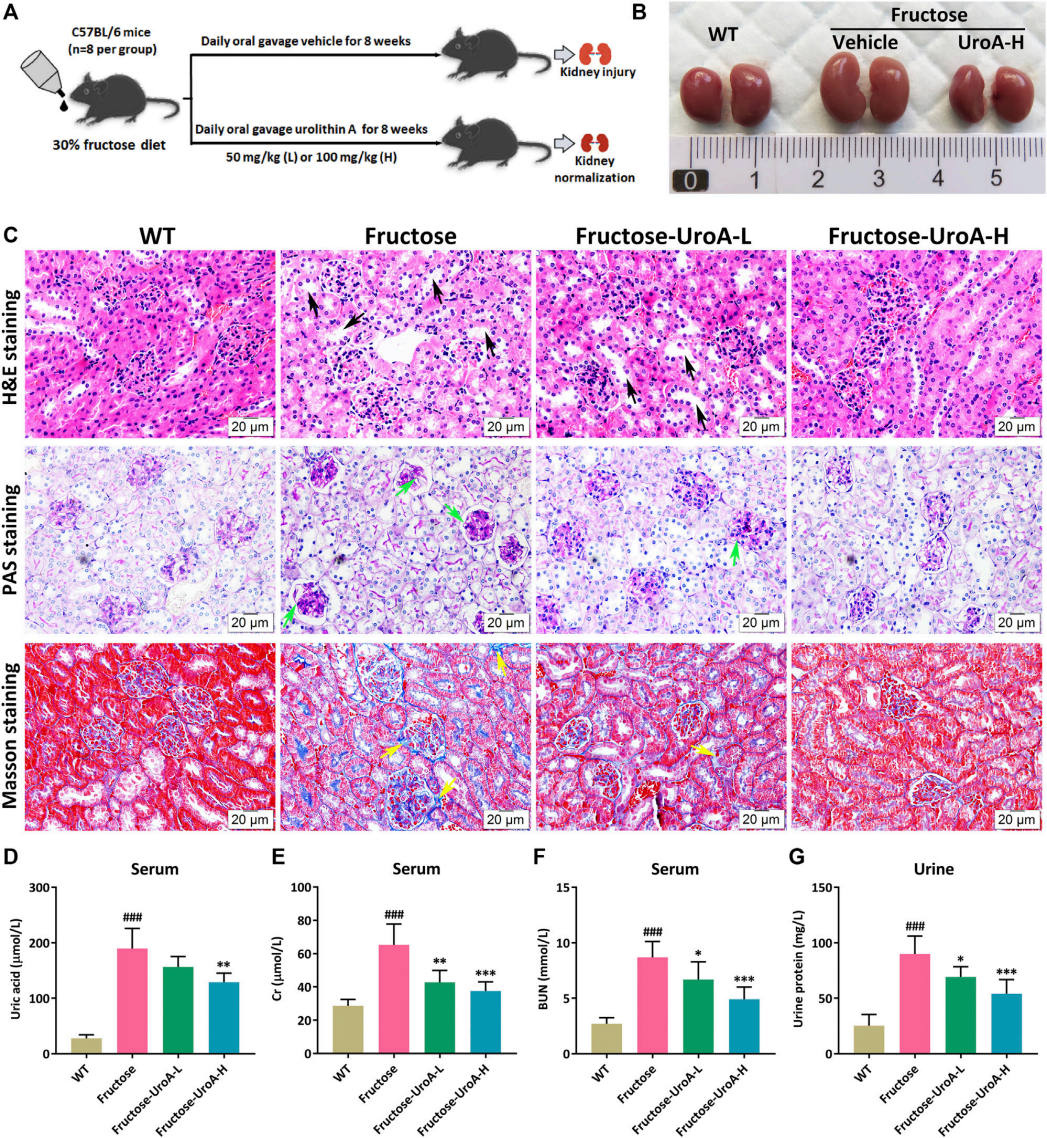
Urolithin A (UroA) ameliorates fructose-induced hyperuricemic nephropathic in mice. **(A)** Study design. **(B)** Kidney macroscopic appearance. **(C)** Hematoxylin-eosin (H&E), Periodic Acid-Schiff (PAS), and Masson’s trichrome staining of kidney tissues from the wild-type (WT) or fructose-fed mice with intragastric administration of either vehicle or UroA, respectively (Scale bar: 20 μm). Fructose-fed mice showed obvious pathological changes in kidney tissues, including tubular hypertrophy and dilation (black arrow), glomerular basement membrane thickening (green arrow), and collagen deposition (yellow arrow). **(D–G)** Fructose-fed mice exhibited a decline in serum uric acid **(D)**, creatinine (Gr) **(E)**, blood urea nitrogen (BUN) **(F),** and urine protein **(G)** after receiving UroA treatment for 8 weeks. Mean ± S.D., *n* = 8. ^###^ *p* < 0.001 *versus* the WT group; ^*^ *p* < 0.05, ^**^ *p* < 0.01, ^***^ *p* < 0.001 *versus* the fructose group. UroA-L and UroA-H represent intragastric administration of urolithin A at low (50 mg/kg/day) and high (100 mg/kg/day) doses, respectively.

Long-term fructose consumption is associated with hyperuricemia, a significant risk factor for chronic kidney disease (CKD) (Gersch et al., 2007). To illustrate the efficacy of UroA improving fructose-induced hyperuricemic nephropathy, UroA intervention (50 or 100 mg/kg) was administrated by gavage to fructose-fed mice for 8 weeks (Figure 1A). Morphologically, kidneys showed mildly swelling in fructose-fed mice while tending to normal after UroA treatment (Figure 1B). Histopathological examination revealed tubular hypertrophy and dilation, glomerular basement membrane thickening, and collagen deposition in the nephropathic mice after 8 weeks of fructose consumption, markedly alleviated by UroA treatment (Figure 1C). Increasing serum uric acid, creatinine (Cr), blood urea nitrogen (BUN), and urinary protein levels are often observed in patients with hyperuricemia-related renal diseases (Sato et al., 2019). Similarly, the content of serum uric acid, Cr, BUN, and urinary protein were markedly higher in fructose-fed mice than that in the WT group but significantly reduced in UroA-treated cohorts (Figures 1D–F). Additionally, the elevation of CKD biomarker kidney injury molecule-1 (KIM-1) in fructose-fed mice was effectively reduced by UroA treatment at both serum content and mRNA level (Supplementary Figure S1). Altogether, these results suggest that UroA effectively ameliorates fructose-induced hyperuricemic nephropathy.

### Urolithin A (UroA) Attenuates Renal Oxidative Stress in Fructose-Induced Urate Nephropathic Mice

**FIGURE 2:**
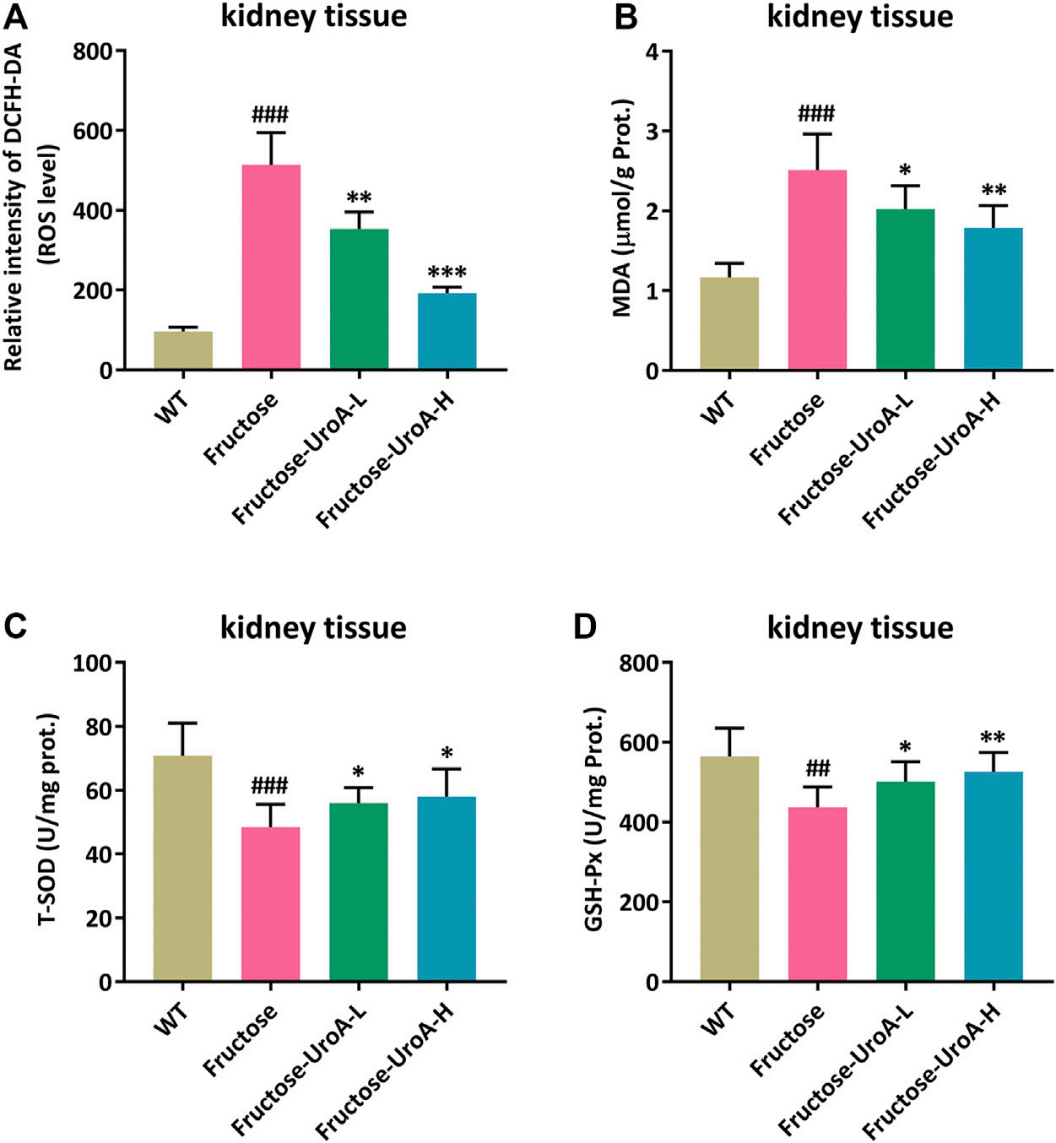
Urolithin A (UroA) attenuates renal oxidative stress in fructose-induced urate nephropathic mice. **(A)** ROS level in renal tissues measured by 2′, 7′-Dichlorodihydrofluorescein diacetate (DCFH-DA) staining. **(B–D)** MDA content and T-SOD/GSH-Px antioxidant enzyme activity in kidney tissues from the wild-type (WT) or fructose-fed mice with intragastric administration of either vehicle or UroA, respectively. Mean ± S.D., *n* = 8. ^##^ *p* < 0.01, ^###^ *p* < 0.001 *versus* the WT group; ^*^ *p* < 0.05, ^**^ *p* < 0.01, ^***^ *p* < 0.001 *versus* the fructose group. UroA-L and UroA-H represent intragastric administration of urolithin A at low (50 mg/kg/day) and high (100 mg/kg/day) doses, respectively. Prot. is the abbreviation of protein.

In this study, reactive oxygen species (ROS) production was prominently elevated in the kidney of fructose-fed nephropathic mice compared with that in mice of WT group, and it was strikingly suppressed by UroA treatment (Figure 2A). MDA, T-SOD, and GSH-Px are common indicators of oxidative stress. The renal content of MDA was increased along with decreased T-SOD and GSH-Px activity in fructose-fed mice *versus* controls. In contrast, UroA significantly decreased the MDA level and enhanced T-SOD and GSH-Px activity in fructose-induced nephropathic mice (Figures 2B–D). These findings demonstrate that UroA can attenuate renal oxidative stress in urate nephropathy.

### Urolithin A (UroA) Impairs STING-NLRP3 Axis Mediated Renal Inflammatory Response *in vivo*

**FIGURE 3:**
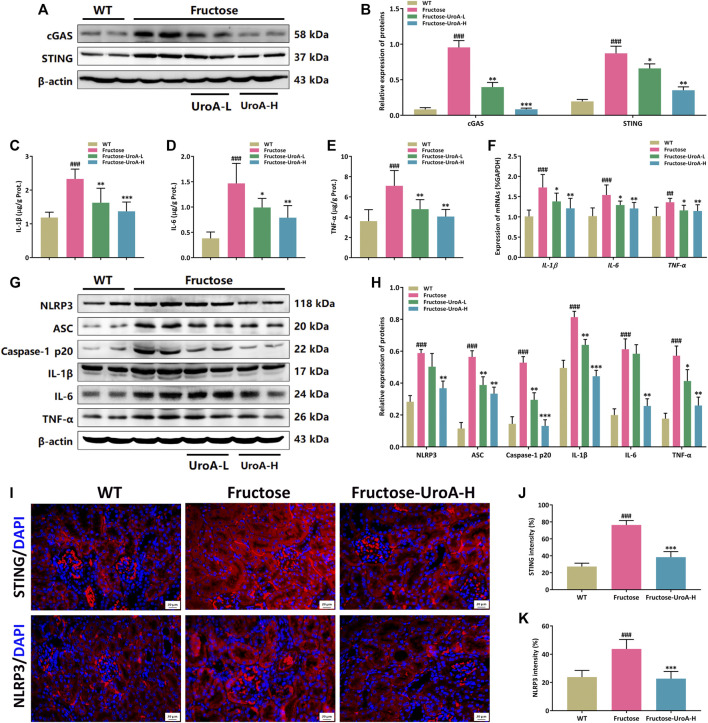
Urolithin A (UroA) impairs STING-NLRP3 axis mediated renal inflammatory response *in vivo*. **(A,B)** Representative immunoblot images of cGAS and STING protein expression in kidneys **(A)** and densitometric analysis **(B)**. **(C–E)** The level of IL-1β **(C)**, IL-6 **(D)**, and TNF-α **(E)** in kidney tissues were detected by commercial enzyme-linked immunosorbent assays (ELISA). **(F)** Histograms show RT-qPCR quantification of *IL-1β*, *IL-6,* and *TNF-*α mRNA expression in the kidney tissues from the wild-type (WT) or fructose-fed mice with intragastric administration of either vehicle or UroA, respectively. The representative immunoblots of NLRP3, ASC, Caspase-1 p20, IL-1β, IL-6, and TNF-α were shown in **(G)**. **(H)** Histograms show the protein expression of NLRP3, ASC, Caspase-1 p20, IL-1β, IL-6, and TNF-α quantified through the western blotting optical analysis shown in **(G)**. β-actin was used as an internal reference. **(I)** Representative images of immunofluorescence staining of STING and NLRP3 of kidney tissue sections. Original magnification: ×400; Scale bar: 20 μm. **(J,K)** Densitometric analysis of STING **(J)** and NLRP3 **(K)**. Mean ± S.D., *n* = 8 for RT-qPCR quantification analysis and *n* = 5 for kidney protein expression analysis. ^##^ *p* < 0.01, ^###^ *p* < 0.001 *versus* the WT group; ^*^ *p* < 0.05, ^**^ *p* < 0.01, ^***^ *p* < 0.001 *versus* the fructose group. UroA-L and UroA-H represent intragastric administration of urolithin A at low (50 mg/kg/day) and high (100 mg/kg/day) doses, respectively. Prot.: protein.

NLRP3 inflammasome is a multi-protein complex composed of NLRP3, apoptosis-associated speck-like protein (ASC), and cysteine aspartate-specific proteases-1 (caspase-1) that serves as a pivotal component on regulating the inflammatory response in CKD development. The cGAS-STING signaling is associated with NLRP3 inflammasome activation (Chung et al., 2019). Here, we investigated the inhibitory effect of UroA on the STING-NLRP3-mediated renal inflammation against fructose-induced hyperuricemic nephropathy. It is intriguing to find that UroA suppressed the renal protein expressions of cGAS and STING in mice (Figures 3A,B). ELISA assay showed that the levels of inflammatory cytokines in both kidney tissue and serum, including IL-1β, IL-6, and TNF-α, were decreased after UroA treatment (Figures 3C–E, Supplementary Figure S2). Meanwhile, the mRNA and protein levels of *IL-1β*, *IL-6*, and *TNF-*α were decreased by UroA administration (Figures 3F–H). In addition, UroA down-regulated the renal protein expressions of NLRP3, ASC, and Caspase-1 p20 in mice (Figures 3G,H). To further insight into the expressions of STING and NLRP3 in the kidney, we performed immunofluorescence, which showed that STING and NLRP3 were activation in the kidney of fructose feeding mice and effectively reduced by UroA administration (Figures 3I–K). These data suggest that UroA effectively alleviates renal inflammatory response and suppresses the STING-NLRP3 axis in nephropathic mice.

### Urolithin A (UroA) Promotes Renal PINK1/Parkin-Mediated Mitophagy in Fructose-Fed Mice

**FIGURE 4:**
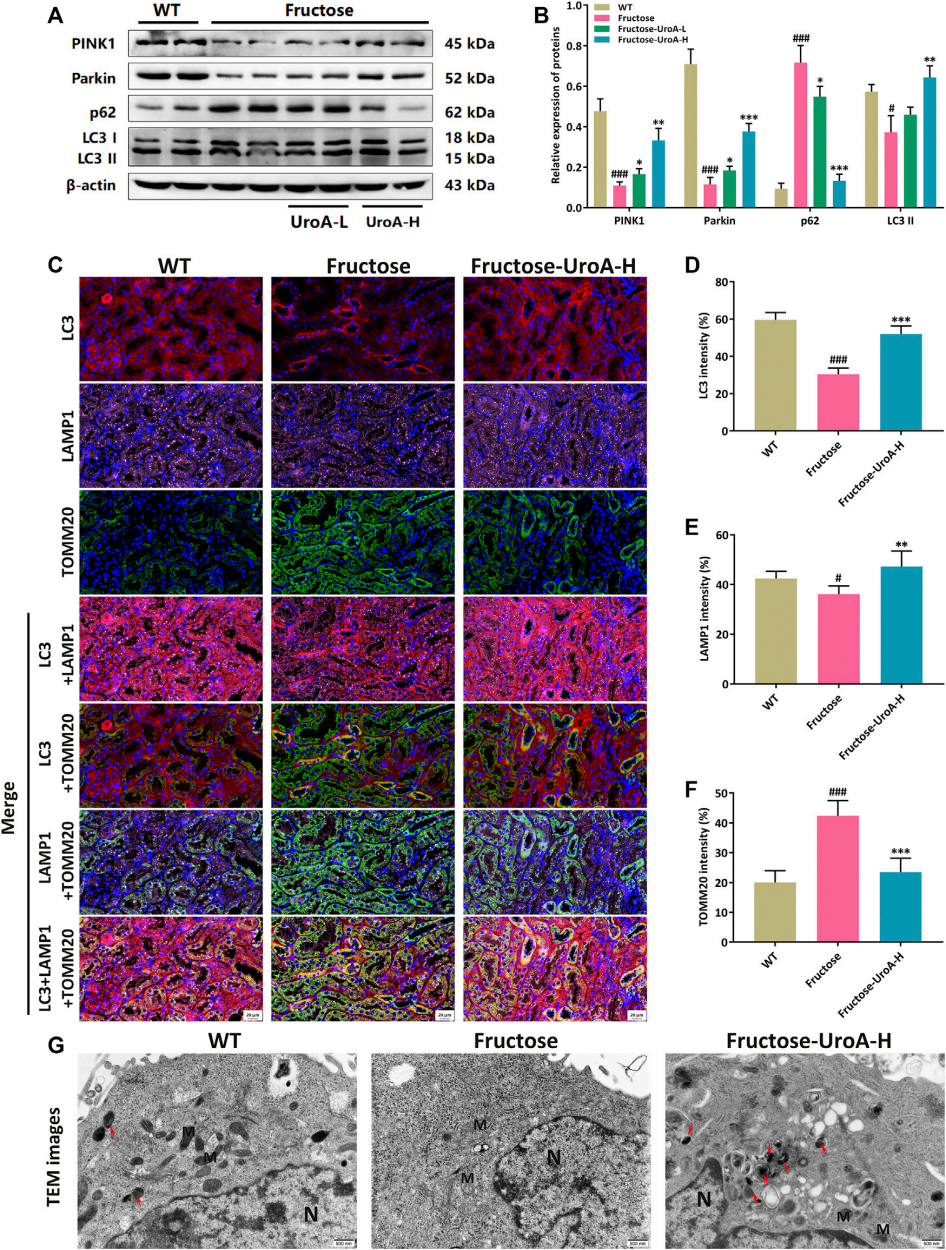
Urolithin A (UroA) promotes renal PINK1/Parkin-mediated mitophagy in fructose-fed mice. **(A,B)** Representative immunoblots of PINK1, Parkin, p62, and LC3 I/II protein levels in mouse kidneys **(A)** and densitometric analysis **(B)**. β-actin was used as an internal reference. **(C)** Representative images of immunofluorescence staining of LC3, LAMP1, and TOMM20 of kidney tissue sections (Red, LC3; Purple, LAMP1; Green, TOMM20). Original magnification: ×400; Scale bar: 20 μm. **(D–F)** Densitometric analysis of LC3 **(D)**, LAMP1 **(E)**, and TOMM20 **(F)**. **(G)** Representative transmission electron microscopy (TEM) images in the kidney tissues from the wild-type (WT) or fructose-fed mice with intragastric administration of either vehicle or UroA, respectively. N: nucleus; M: mitochondria; autolysosomes (red arrow). Mean ± S.D., *n* = 5. ^#^ *p* < 0.05, ^###^ *p* < 0.001 *versus* the WT group; ^*^ *p* < 0.05, ^**^ *p* < 0.01, ^***^ *p* < 0.001 *versus* the fructose group. UroA-L and UroA-H represent intragastric administration of urolithin A at low (50 mg/kg/day) and high (100 mg/kg/day) doses, respectively.

As a quality control mechanism for the clearance of damaged mitochondria, mitophagy has been reported to participate in the development of diverse metabolic diseases, including nephropathy (Livingston et al., 2019). In this study, the protein expressions of PINK1, Parkin, and LC3 II were increased by contrast with the decreased of p62 in the kidneys of UroA-treated mice (Figures 4A,B). To further investigate the localization of PINK1/Parkin-mediated mitophagy in the kidneys, we next examined the colocalization of Parkin and translocase of outer mitochondrial membrane 20 (TOMM20), a critical mitochondrial membrane protein as a biomarker of mitochondria. Colocalizations were decreased in the kidneys of fructose-fed mice but were more co-localized after UroA administration (Supplementary Figure S3). According to the mitophagy pathways, damaged mitochondria were transported to lysosomes for degradation *via* LC3 binding. Thus, we further explored the renal colocalization of LC3, TOMM20, and lysosomal-associated membrane protein 1 (LAMP1), three markers of the mitophagy process. It resulted in fewer TOMM20 puncta but more LC3, LAMP1 puncta, and more colocalizations of these three mitophagy markers in the kidneys of UroA-treated mice compared with that in the fructose-fed mice (Figures 4C–F), indicating the occurrence of mitophagy process. Furthermore, transmission electron microscopy (TEM) assessment showed that mitochondria in renal tubular epithelial cells were swollen, and the ridge structure almost disappeared, with no obvious mitophagy observed in nephropathic mice. Of note, UroA administration restored the impaired renal mitophagy in fructose-fed mice (Figure 4G). Altogether, our data indicate that UroA facilitates PINK1/Parkin-mediated mitophagy in the kidneys of nephropathic mice.

### Urolithin A (UroA) Inhibits STING-NLRP3 Activation by Promoting Parkin-Dependent Mitophagy *in vitro*

**FIGURE 5:**
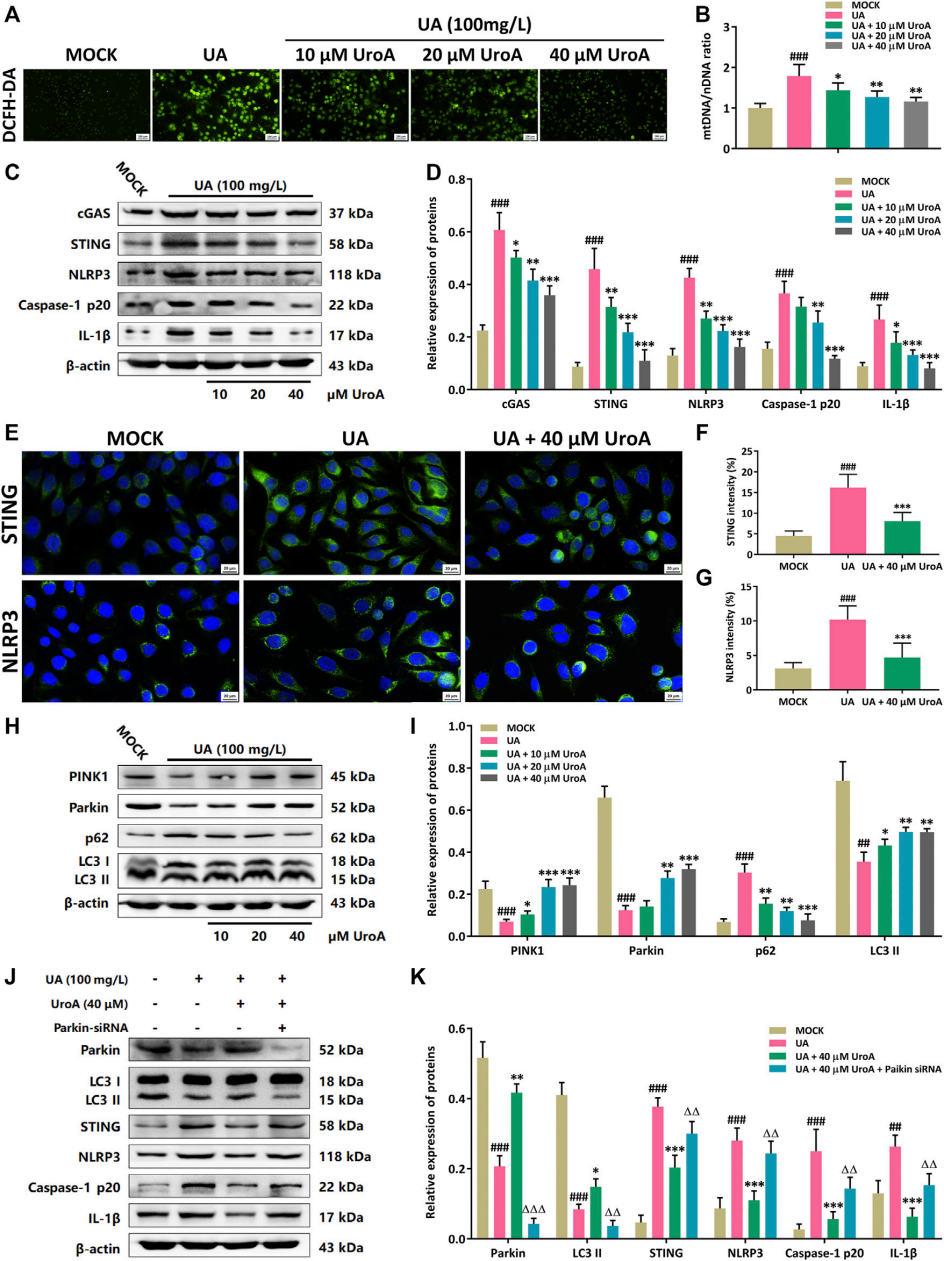
Urolithin A (UroA) inhibits STING-NLRP3 activation by promoting Parkin-dependent mitophagy *in vitro*. **(A)** Representative immunofluorescence blots of ROS levels in the MOCK or uric acid (UA)-induced HK-2 cells with the administration of UroA (0–40 μM), respectively. **(B)** Mitochondrial DNA (mtDNA) to nuclear DNA (nDNA) ratio in uric acid-induced HK-2 cells with the administration of UroA. **(C,D)** Representative immunoblots of cGAS, STING, NLRP3, Caspase-1 p20, and IL-1β in HK-2 cells **(C)** and densitometric analysis **(D)**. **(E–G)** Representative blots of STING and NLRP3 immunofluorescence in HK-2 cells **(E)** and fluorescence intensity analysis **(F,G)**. **(H,I)** Representative immunoblots of PINK1, Parkin, p62, and LC3 I/II protein levels in HK-2 cells **(H)** and densitometric analysis **(I)**. **(J,K)** Representative immunoblots of Parkin, LC3 I/II, STING, NLRP3, Caspase-1 p20, and IL-1β in the uric acid (UA)-induced HK-2 cells after parkin silencing **(J)** and densitometric analysis **(K)**. β-actin was used as an internal reference. Mean ± S.D., *n* = 5. ^##^ *p* < 0.01, ^###^ *p* < 0.001 *versus* the MOCK group; ^*^ *p* < 0.05, ^**^ *p* < 0.01, ^***^ *p* < 0.001 *versus* the UA treated group; ^△△^ *p* < 0.01, ^△△△^ *p* < 0.001 *versus* the UA and UroA co-processing group.

Next, a uric acid (UA)-induced hyperuricemic HK-2 cell model was established for mechanism validation in the efficacy of UroA on ameliorating hyperuricemic nephropathy. Firstly, the ROS level was examined to show that the ROS level was increased in UA-induced cells while it was markedly decreased after UroA intervention (Figure 5A). Since the leakage of mitochondrial DNA (mtDNA) is a significant inducer of activating STING signal, we next determined the mtDNA/nuclear DNA (mtDNA/nDNA) ratio, and it downregulated after UroA intervention (Figure 5B). Western blotting analysis revealed that UroA intervention reduced the protein expression of cGAS, STING, NLRP3, Caspase-1 p20, and IL-1β (Figures 5C,D). Immunofluorescence assay showed that the expression of STING and NLRP3 in UA-induced HK-2 cells was restrained by UroA treatment (Figures 5E–G). Moreover, UroA up-regulated the protein expression of PINK1, Parkin, and LC3 II, and reduced the expression of p62 (Figures 5H,I). We further elucidate the correlation between Parkin-mediated mitophagy and STING-NLRP3 signaling. The results demonstrated that UroA-mediated suppression of STING-NLRP3 cascade was counteracted in Parkin-silenced HK-2 cells (Figures 5J,K). Overall, it indicates that UroA inhibits STING-NLRP3 mediated inflammatory activation by promoting Parkin-dependent mitophagy.

## Discussion

The kidney is an essential hub regulating circulating uric acid since it excretes about 70% of total body uric acid (Lipkowitz, 2012). As the final metabolic production of purine metabolism, excessive uric acid accumulates when the balance between synthesis and excretion is disrupted, leading to hyperuricemia and urate crystal deposition in the kidney, which further facilitates renal oxidative stress and inflammatory response in hyperuricemic nephropathy (Kang and Nakagawa, 2005). A chronic fructose diet is a risk factor for hyperuricemic nephropathy. Urolithin A (UroA) is one of the major natural intestinal metabolites of ellagitannins found in pomegranates, berries, nuts, etc., with various pharmacological potentials (Cheng et al., 2021; Zhang et al., 2022). However, the beneficial effect of UroA against hyperuricemic nephropathy and the underlying mechanism needs further investigation. In this study, we investigated the ameliorative effect of UroA in fructose-induced hyperuricemic nephropathy. Our results revealed that the serum uric acid level and renal function indicators (Cr, BUN, and urine protein) were increased in fructose-fed mice, along with apparent renal injury (tubular hypertrophy and dilation, glomerular basement membrane thickening, and collagen deposition). Significantly, these abnormities were improved after UroA treatment. Mechanistically, UroA inhibited the activation of STING-NLRP3 signaling by promoting PINK1/Parkin-mediated mitophagy. To the best of our knowledge, this study is the first to demonstrate the efficacy of UroA in fructose-induced hyperuricemic nephropathy, and the underlying mechanism may refer to the inhibition of STING-NLRP3 activation and restoration of PINK1/Parkin-mediated mitophagy.

It is also believed that the kidney behaves as a mitochondrial-enriched and metabolically active organ to be a significant consumer of molecule oxygen in the body. The deposition of uric acid and urinary crystals generated by fructose overloading can stimulate excess ROS production, which triggers mitochondrial stress and aberrant tubular inflammation leading to renal injury (Cristóbal-García et al., 2015; Srivastava et al., 2018). Our data revealed that UroA reduced renal ROS and MDA levels accompanied by the elevations of anti-oxidant enzyme activity (t-SOD and GSH-px), which contributes to its anti-oxidant effect in fructose-fed mice. PINK1/Parkin-mediated mitophagy has been indicated as a crucial player in various pathophysiological processes by selective elimination of damaged mitochondria. In detail, PINK1 proteolysis is suppressed when the subset of mitochondria is damaged, allowing the deposition of PINK1 on the outer mitochondrial membrane (OMM) as the prerequisite for recruitment of E3 ubiquitin ligase Parkin (Chen and Dorn, 2013). Subsequently, Parkin ubiquitylates mitochondrial substrates to mediate the formation of polyubiquitin chains, which are further identified by the ubiquitin-binding adaptor p62 (also known as sequestosome 1, SQSTM1) to initiate mitophagy *via* LC3 binding, thereby contributing to the degradation of damaged mitochondria cargo in lysosomes (Geisler et al., 2010; Nguyen et al., 2016). Notably, cellular mitophagy is widely identified in CKD progression as a protective mechanism validated in fructose-induced renal injury (Gu et al., 2021; Ding et al., 2022), indicating that the enhancement of mitophagy that maintains mitochondrial homeostasis can serve as a potential strategy for ameliorating CKD. Mechanistically, we found that UroA increased the protein expressions of PINK1, Parkin, and LC3II to promote mitophagy in fructose-induced nephropathic mice and uric acid-induced *in vitro* experiments. As a selective form of autophagy, the process of mitophagy also mainly consists of three sequential steps, including autophagosome formation, transportation to the lysosomes, and degradation in the lysosomes. LC3, TOMM20, and LAMP1 are chosen as the selective markers for the above three steps, respectively. Hence, we subsequently determined the presence and colocalization to mark the occurrence of mitophagy. Complete results of colocalization revealed that UroA facilitated the transportation of LC3 into mitochondria and afterward fusion with the lysosome, thus promoting cellular mitophagy. All data suggests that UroA is capable of promoting PINK1/Parkin-mediated mitophagy in CKD development.

STING is a crucial regulator of innate immune and inflammatory response, and its activation involved in the process of renal injury (Allison, 2019; Chung et al., 2019). Cyclic GMP-AMP synthase (cGAS) is a cytosolic DNA sensor involved in the development of multiple diseases by binding mtDNA (leakage by mitochondrial injury) directly and catalyzing the production of cyclic GMP-AMP (cGAMP) to activate STING signaling and build the linkage between mitophagy and STING-mediated inflammation (Sun et al., 2013; Willemsen et al., 2021). Considering the interaction between mtDNA and STING activation, we evaluated whether UroA affected the mtDNA/nDNA ratio in uric acid-induced HK-2 cells. Our data suggested that UroA reduced the relative level of mtDNA copy numbers. Moreover, the dysregulated STING signaling recruits TANK-binding kinase 1 (TBK1) to activate the pivotal inflammatory sensor NLRP3 that contributes to the active transformation of proinflammatory cytokines (e.g., IL-1β, IL-18, and MCP-1) (Gaidt et al., 2017; Fischer et al., 2021). Thus, we further investigated whether UroA inhibited STING-NLRP3 axis-mediated inflammatory response. In this study, UroA significantly suppressed the renal protein expressions of STING-NLRP3 signaling and the release of inflammatory factors (IL-1β, IL-6, TNF-α) in fructose-fed mice. There have been reports that STING is presumably activated in macrophages (Luo et al., 2018). Interestingly, we observed STING activation in kidneys of hyperuricemic nephropathy mice, which was decreased after UroA intervention. With that, we further validated the underlying mechanism of UroA in attenuating hyperuricemic nephropathy in uric acid-induced HK-2 cells, and similar results were reconfirmed *in vitro*. Recent studies suggest that PINK1/Parkin-mediated mitophagy attenuates renal inflammatory injury by inhibiting cGAS-STING signaling activation (Sliter et al., 2018; Chung et al., 2019). Similarly, our data showed that the inhibitory effect of UroA in STING-NLRP3 activation was impaired after silencing Parkin in HK-2 cells, indicating that UroA may abolish STING-NLRP3 axis-mediated inflammation by retriggering Parkin-dependent mitophagy. Additionally, our data also revealed that UroA repressed the expression of fibrotic markers (TGF-β and α-SMA), demonstrating that UroA could be beneficial for improving renal fibrosis (Supplementary Figure S4) in CKD progression.

In summary, our study demonstrates the ameliorative effect of UroA in fructose-induced hyperuricemic nephropathy. *In vivo* and *in vitro* data suggest that UroA can mitigate STING-NLRP3 axis-mediated inflammatory response by promoting Parkin-dependent mitophagy. Therefore, dietary supplementation with UroA or ellagitannins-rich foods might serve as a promising approach to delaying CKD progression.

## Data Availability

The original contributions presented in the study are included in the article/Supplementary Material, further inquiries can be directed to the corresponding authors.

## Abbreviations

BUN, blood urea nitrogen; Caspase-1, cysteinyl aspartate specific proteinase-1; cGAS, cyclic GMP-AMP synthase; cGAMP, cyclic GMP-AMP; CKD, chronic kidney disease; Cr, creatinine; DCFH-DA, 2,7-Dichlorodi-hydrofluorescein diacetate; IL-1β, interleukin 1β; IL-6, interleukin 6; KIM-1, kidney injury molecule-1; LAMP1, lysosomal-associated membrane protein 1; mtDNA, mitochondrial DNA; ND1, NADH dehydrogenase subunit 1; NLRP3, NOD-like receptor protein 3; ROS, reactive oxygen species; STING, stimulator of interferon genes; TBK1, TANK-binding kinase 1; TNF‐α, tumor necrosis factor α; TOMM20, translocase of outer mitochondrial membrane 20; UA, uric acid; UroA, urolithin A; WT, wild-type.
