# Supplementary material for: Urolithin A Attenuates Hyperuricemic Nephropathy in Fructose-Fed Mice by Impairing STING-NLRP3 Axis-Mediated Inflammatory Response via Restoration of Parkin-Dependent Mitophagy
Source: Front Pharmacol. 2022 Jun 15;13:907209. doi: 10.3389/fphar.2022.907209 (PMC9240289; doi:10.3389/fphar.2022.907209)
Supplement: Supplementary file 1 [file DataSheet1.DOCX]

Supplementary Material

Urolithin A attenuates hyperuricemic nephropathy in fructose-fed mice by impairing STING-NLRP3 axis-mediated inflammatory response *via* restoration of Parkin-dependent mitophagy

**Supplementary Figures**

**Supplementary Figure 1. Urolithin A (UroA) reduces serum KIM-1 level and mRNA expression in kidney of fructose-fed mice.** Mean ± S.D., *n* = 8. ^##^*P* < 0.01, ^###^*P* < 0.001 *versus* the WT group; ^*^*P* < 0.05, ^**^*P* < 0.01, ^***^*P* < 0.001 *versus* the fructose group. UroA-L and UroA-H represent intragastric administration of urolithin A at low (50 mg/kg/day) and high (100 mg/kg/day) doses, respectively.


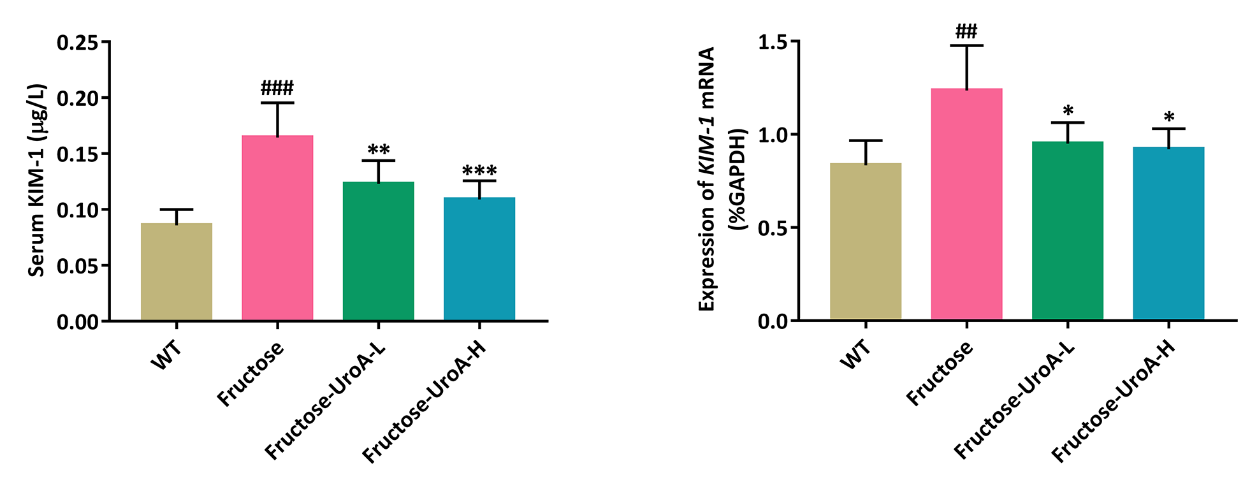


**Supplementary Figure 2. Urolithin A (UroA) reduces serum IL-1β, IL-6, and TNF-α levels in fructose-fed mice.** Histograms show the serum levels of key inflammatory factors (IL-1β, IL-6, and TNF-α) quantified by ELISA assay in fructose-fed mice. Mean ± S.D., *n* = 8. ^###^*P* < 0.001 *versus* the WT group; ^**^*P* < 0.01, ^***^*P* < 0.001 *versus* the fructose group. UroA-L and UroA-H represent intragastric administration of urolithin A at low (50 mg/kg/day) and high (100 mg/kg/day) doses, respectively.


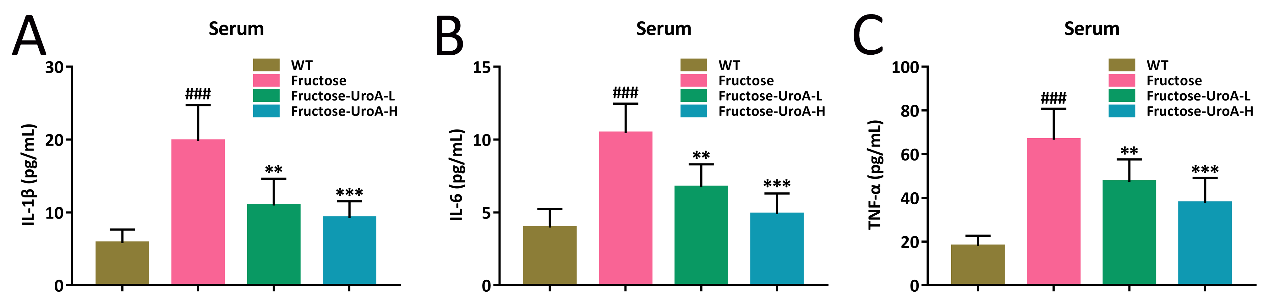


**Supplementary Figure 3. Urolithin A (UroA) promotes the colocalization of Parkin-TOMM20 in the kidney.** To further investigate the localization of PINK1/Parkin-mediated mitophagy in kidneys, we next examined the colocalization of Parkin and TOMM20, an important mitochondrial membrane protein as a biomarker of mitochondria. Their colocalizations were decreased in the kidneys of fructose-fed mice but were more co-localized after UroA administration. UroA-L and UroA-H represent intragastric administration of urolithin A at low (50 mg/kg/day) and high (100 mg/kg/day) doses, respectively.


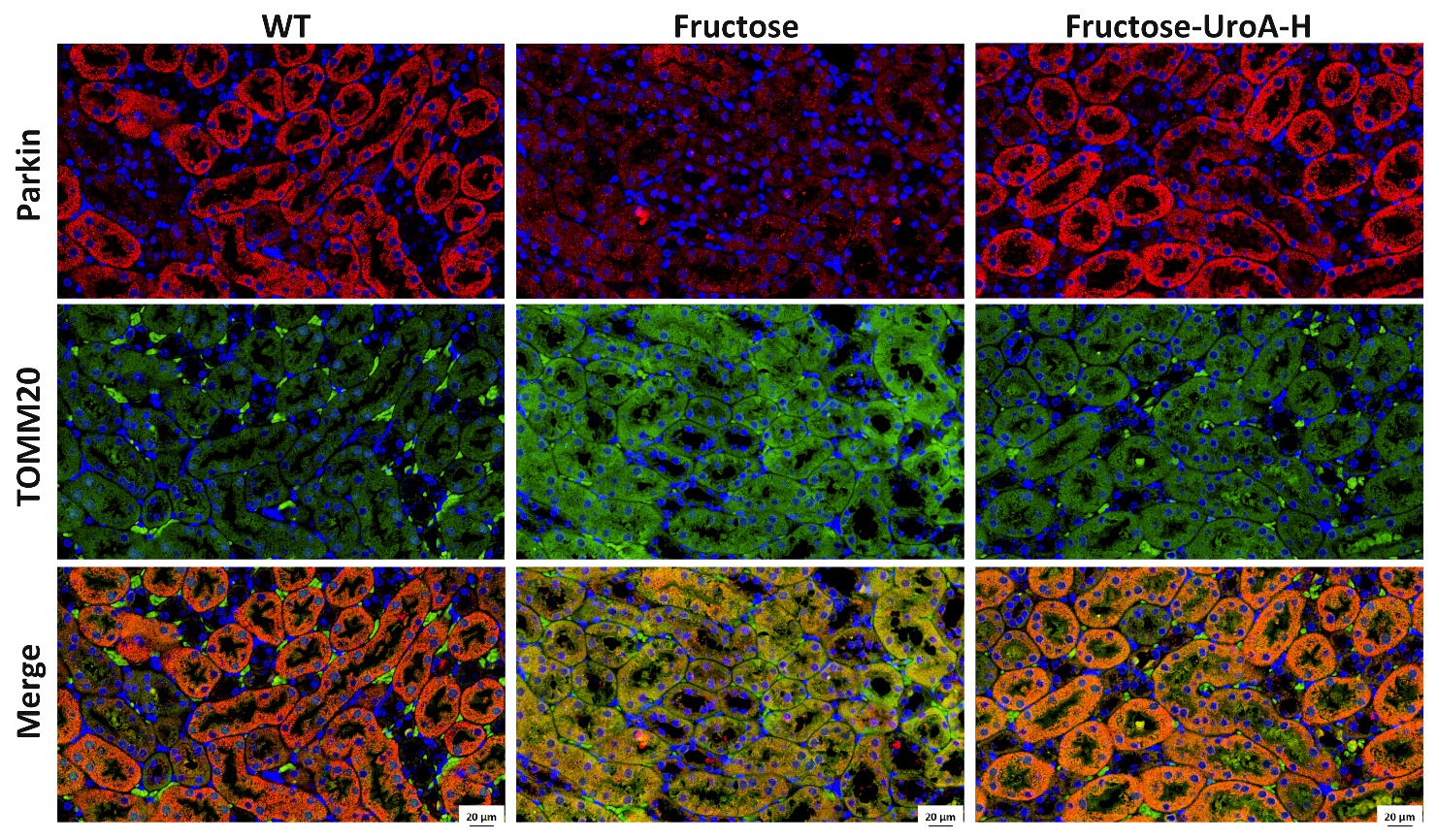


**Supplementary Figure 4. Urolithin A (UroA) represses the protein expressions of TGF-β1 and α-SMA in nephropathic mice.** **(A, B)** Representative immunoblots of TGF-β1 and α-SMA protein levels in mouse kidneys (A) and densitometric analysis (B). **(C)** Representative images of immunohistochemical staining of TGF-β1 and α-SMA of kidney tissue sections. Original magnification: 400×; Scale bar: 20 μm. Mean ± S.D., *n* = 5. ^###^*P* < 0.001 *versus* the WT group; ^**^*P* < 0.01, ^***^*P* < 0.001 *versus* the fructose-fed group. UroA-L and UroA-H represent intragastric administration of urolithin A at low (50 mg/kg/day) and high (100 mg/kg/day) doses, respectively.


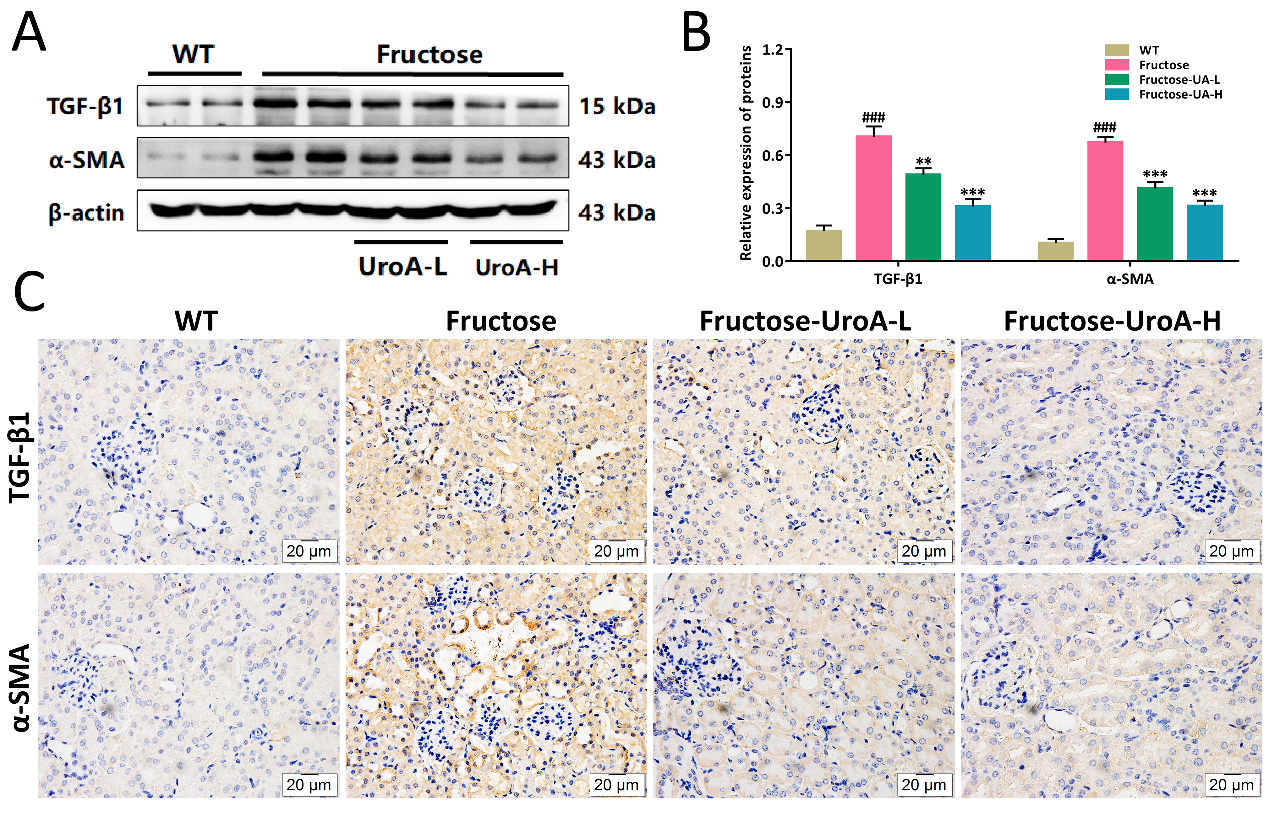


**Supplementary Figure 5.** The effects of urolithin A (UroA) on cell viability of HK-2 cell lines.


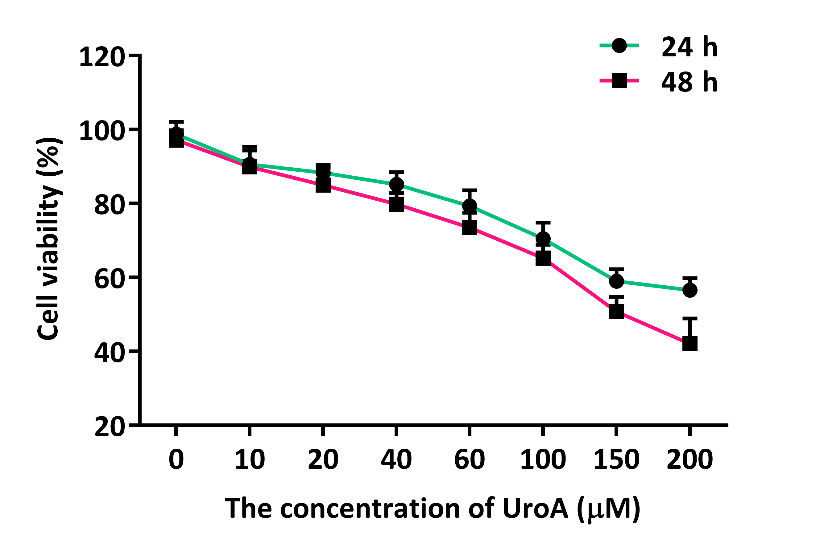


**Supplementary Tables**

**Supplementary Table I.** Sequences of primers used in real-time quantitative PCR (RT-qPCR).

| **Gene** | **Sequences (5’ to 3’)** |
| --- | --- |
| *Mus-GAPDH* | Forward: TCAATGAAGGGGTCGTTGAT  Reverse: CGTCCCGTAGACAAAATGGT |
| *Mus-KIM-1* | Forward: CAAGTTAAACCAGAGATTCCCAC  Reverse: CGTGATGCTGAGAAGTCTCA |
| *Mus-IL-1β* | Forward: GAAATGCCACCTTTTGACAGTG  Reverse: TGGATGCTCTCATCAGGACAG |
| *Mus-IL-6* | Forward: TAGTCCTTCCTACCCCAATTTCC  Reverse: TTGGTCCTTAGCCACTCCTTC |
| *Mus-TNF-α* | Forward: CAGGCGGTGCCTATGTCTC  Reverse: CGATCACCCCGAAGTTCAGTAG |
| *Homo-GAPDH* | Forward: ACCACAGTCCATGCCATCAC |
|  | Reverse: TCCACCACCCTGTTGCTGTA |
| *Homo-ND1* | Forward: CACTTTCCACACAGACATCA |
|  | Reverse: TGGTTAGGCTGGTGTTAGGG |

**Supplementary Table II.** List of the primary antibodies used for Western blot analysis (WB), immunohistochemistry (IHC), and immunofluorescence (IF).

| **Protein** | **Antibody (and catalog number)** | **Application** |
| --- | --- | --- |
| ASC | Rabbit polyclonal (A1170) | WB ^ |
| Caspase-1 p20 | Rabbit monoclonal (89332) | WB † |
| cGAS | Rabbit polyclonal (26416-1-AP) | WB # |
| IL-1β | Rabbit polyclonal (TA5103) | WB § |
| IL-6 | Rabbit monoclonal (14351-1-AP) | WB # |
| LAMP1 | Rabbit polyclonal (A2582) | IF ^ |
| LC3 I/II | Rabbit polyclonal (14600-1-AP) | WB; IF # |
| NLRP3 | Rabbit polyclonal (T55651) | WB; IF § |
| p62 | Rabbit monoclonal (A19700) | WB; IF ^ |
| Parkin | Rabbit polyclonal (14060-1-AP) | WB, IF # |
| PINK1 | Rabbit polyclonal (23274-1-AP) | WB # |
| STING | Rabbit monoclonal (66680-1-Ig) | WB # |
| TGF-β1 | Rabbit polyclonal (WL02193) | WB; IHC * |
| TNF-α | Rabbit polyclonal (17590-1-AP) | WB # |
| TOMM20 | Rabbit polyclonal (11802-1-AP) | IF # |
| α-SMA | Rabbit polyclonal (WL02501) | WB; IHC * |
| β-actin | Mouse monoclonal (66009-1-Ig) | WB # |

† Provided by Cell Signaling Technology Inc. (Danvers, MA).

# Provided by Proteintech (Wuhan, China)

* Provided by Wanleibio (Shenyang, China)

^ Provided by ABclonal (Wuhan, China)

§ Provided by Abmart (Shanghai, China)
